# Patient characteristics and testing over COVID-19 waves 1 and 2 from the first German COVID-19 testing unit in Munich, Germany

**DOI:** 10.1186/s12879-023-08068-4

**Published:** 2023-02-23

**Authors:** Harinee Srinivasan, Hannah Tuulikki Hohl, Christian Heumann, Guenter Froeschl

**Affiliations:** 1 Center for International Health, Ludwig-Maximilians-Universität, Ziemssenstr. 5, 80336 Munich, Germany; 2 Division of Infectious Diseases and Tropical Medicine, University Hospital, LMU Munich, Leopoldstr. 5, 80802 Munich, Germany; 3 Department of Statistics, University of Munich (LMU), Ludwigstr. 33, 80539 Munich, Germany

**Keywords:** SARS-CoV-2, COVID-19 testing unit, Public health measures, Munich, Germany

## Abstract

**Background:**

In Munich, the first German case of severe acute respiratory syndrome coronavirus 2 (SARS-CoV-2) was detected on 27 January 2020 at the Division of Infectious Diseases and Tropical Medicine of the University Hospital LMU Munich (DIDTM), and consecutively the Covid Testing Unit was established. Germany advocated several public health measures to control the outbreak. This study investigates the effects of measures on health service utilization in the public, which in turn can alter case numbers and test positivity rates.

**Method:**

Our retrospective observational study was conducted to determine the effects of public health measures on the utilization of a testing facility and positivity rates from the first operational COVID-19 testing facility in Munich for waves 1 and 2 over a period of 14 months. This was accomplished by comparing trends in client characteristics including age, gender, symptoms, and socio-demographic aspects over time to non-pharmaceutical measures in Germany. To depict trend changes in testing numbers over time, we developed a negative binomial model with multiple breakpoints.

**Results:**

In total 9861 tests were conducted on 6989 clients. The clients were mostly young (median age: 34), female (60.58%), and asymptomatic (67.89%). Among those who tested positive for SARS-CoV-2, 67.72% were symptomatic while the percentage was 29.06% among those who tested negative. There are other risk factors, but a SARS-CoV-2-positive colleague at work is the most prominent factor. Trend changes in the clients’ testing numbers could be attributed to the implementation of various public health measures, testing strategies, and attitudes of individuals toward the pandemic. However, test positivity rates did not change substantially during the second wave of the pandemic.

**Conclusion:**

We could show that implementation or changes in public health measures have a strong effect on the utilization of testing facilities by the general public, which independently of the true epidemiological background situation can result in changing test numbers.

**Supplementary Information:**

The online version contains supplementary material available at 10.1186/s12879-023-08068-4.

## Introduction

The COVID-19 pandemic that began in late 2019 has caused unprecedented global disruption [1]. It has challenged both national and supra-national health systems.

Although the overall global case fatality rate, which was reported at 2.2% until December 29, 2020 [2], can be considered to be relatively low compared to other infectious diseases [3], the global spread of infections has led to high absolute numbers of fatalities. Globally, there have been more than 6.3 million reported deaths and 562 million cases confirmed up to 18 July 2022 [4]. The causative agent, SARS-CoV-2, has become one of the most important subjects of research, be it in terms of virological aspects, diagnostics, prevention, or treatment. This also leads to a diversion of resources from other health issues toward COVID-19 [5].

Early in the pandemic, Europe emerged as a focal point in the global spread of infections, with Germany having its first cases imported in January 2020 [6]. The state of Bavaria, which is in the southeast of Germany, was one of the most rapidly and intensely affected federal states during the initial pandemic wave [7, 8]. Beginning on 27 January 2020, the first German cluster of COVID-19 was detected at the Division of Infectious Diseases and Tropical Medicine at the University Hospital, LMU Munich. As an initial response to this local outbreak among employees of the car part manufacturing company “Webasto“ located south of Munich [9], the COVID-19 Testing Unit (CTU) Munich was founded, which served as a significant contact point for the containment of the pandemic from its onset. Later the target groups were changed to people with specific risk profiles or occupational groups, such as healthcare workers.

Lockdowns have been implemented in a number of nations to combat the current coronavirus outbreak [10]. Although the number and types of measures used by governments and decision-makers vary, they frequently involve contact limitations, for example through the closure of numerous public gathering areas, such as schools, government offices, and shopping malls [11]. The interactions between different measures are manifold and context-dependent. In addition, the reported case numbers greatly depend on accessibility, acceptability, and ultimately utilization of testing offers. Thus, it has to be assumed that officially reported case numbers only indirectly reflect the true picture of virus circulation in a given population. In order to highlight the potential effects of different public health measures in the specific context of the city of Munich, we sought to correlate client data from the CTU Munich including client characteristics, test numbers, and positivity rates with various public health measures across the 14-month running period of the CTU, which agglomerated waves 1 and 2.

## Methodology

### Study design

This study is a retrospective observational study using client records of the first German testing unit, operated by the University Hospital of the Ludwig-Maximilians-Universität in Munich, Germany.

### Objectives

The objective of this study was to identify the potential impact of public health measures on the utilization and test positivity rate by the example of CTU Munich. In addition, we want to inform decision-makers and operators of comparable units about the substantial changes in client characteristics over time, which are influenced by public health measures, and in turn have an impact on utilization and positivity rates.

### Study setting

This study comprises data collected from symptomatic patients and non-symptomatic clients (henceforth collectively referred to as ‘clients’) that were tested for infection with SARS-CoV-2 at the CTU at the Division of Infectious Diseases and Tropical Medicine of the University Hospital LMU Munich (DIDTM), in the capital of the state of Bavaria in southern Germany. The first German patient that tested positive was admitted to the outpatient department of the DIDTM on 27 January 2020. On 28 January 2020, the CTU was established. On 23 March 2020, the CTU was transferred into a tent structure in front of the main building of the DIDTM. The CTU tent was the first and longest-running structure in Germany at the time. The unit consisted of the beginning of a vacant tract of the DIDTM in the city center of Munich and was in March 2020 replaced by a tent structure in front of the DIDTM in a city street that was closed down for this purpose. Fees were not directly collected but charged from the respective public or private health insurance. At a later stage, the state of Bavaria took over all testing costs. The CTU served as a testing structure in the control measures of the first German COVID-19 cluster. Later, the CTU was opened to general citizens, but with a focus on health care workers. The CTU remained operational until 26 March 2021.

### Study population

Across the operating time of the CTU, the constitution of the study population changed, including persons with indicative symptoms, those with indicative exposures, persons belonging to certain occupational risk groups, but also persons without indicative characteristics. Clients were referred to the CTU by occupational health physicians at their place of work, by school administrations, or were self-referred.

### Data collection

Data were obtained over 14 months (between 27 January 2020 and 26 March 2021). The independent variables are patient characteristics such as socio-demographic data, clinical data, and exposure and travel history. In addition, data were collected on public health measures through literature and document review. The outcome variables are test numbers and test results. Client characteristics on age, gender, exposures (travel, workplace), and symptoms were collected using self-completed case report forms, which were initially paper-based and since 01 February 2021, online-based. Samples were collected as deep nasopharyngeal swabs. Where the nasopharyngeal sample pathway was not possible, a deep oropharyngeal swab was taken. Swabs were sent to affiliated laboratories in Munich which included the Institute for Microbiology of the Armed Forces, the Max-von-Pettenkofer-Institute of the Ludwig-Maximilians-Universität, and “Labor Becker & Kollegen”. Analysis was conducted by real-time polymerase chain reaction. The client’s exposure status was determined based on interaction with a COVID-19 positive patient. “Employee exposure “at work refers to non-healthcare professionals getting in contact with a COVID-19 case “Exposure to a patient at work” refers to healthcare employees’ encounters with COVID-19 patients in hospitals and nursing homes. “Private exposure “ refers to clients reporting exposure to a COVID-19 case in a private domain. Travel exposures relate to recent travel from a risk area.

### Data entry and analysis

All data were manually transferred to a Microsoft Excel spreadsheet database. Before the transfer of the database to the research team, the data was irreversibly anonymized. Descriptive presentation of data is executed using proportions, median and interquartile range for non-normally distributed data.

We used a regression model with breakpoints for client numbers per seven-day moving average to determine the course of client numbers over time. The daily numbers of CTU clients had a high variation between e.g. different days of the week, therefore we decided to use 7-day moving averages to smooth these effects. The negative binomial model has been chosen because the 7-day moving average numbers are correlated and this model can adjust for that using an additional over dispersion parameter. Breakpoints are supportive in identifying critical time points in the dynamic presentation of continuous data and allow comparison and contextualization with environmental events. The Bayesian Information Criterion was used to calculate the desirable number of breakpoints. A negative binomial model was chosen by R^2 value for final analysis.

General statistical analysis was performed using STATA software version 17.0 and breakpoint analysis with R version 4.2.1, software package “segmented” version 1.6-0.

## Results

### Test numbers and test results by client socio-demographics and clinical characteristics

Overall, 9861 SARS-CoV-2 RT-PCR tests were performed at the CTU from 27 January 2020, until 26 March 2021, among 6989 clients tested. Amongst the RT-PCR test results, 80 had to be excluded due to invalid results, leaving 9781 RT-PCR tests in 6989 clients for further analysis.

Of the 6989 clients tested 4234 were female (60.58%) and 2754 (39.40%) were male clients, with one person not reporting on gender. A notably higher proportion of women (31.76%) were symptomatic than men (27.37%) (p-value < 0.01). The test positivity rate was marginally but non-significantly higher in female clients (n = 173, 66.03%) than in male clients (n = 89, 33.97**%**). Out of the age groups reported, group 20–39 years of age showed the highest percentage of positive results (n = 128, 48.85%) (see Table 1).

**Table 1:** Client socio-demographics and test-positivity at the CTU

Client characteristics	Tested negative	Tested positive	p-value
**Total**	**9519**	**262**	
**Age**			0.01
0–19	943 9.91%	14 5.34%	
20–39	4929 51.81%	128 48.85%	
40–59	2984 31.37%	105 40.08%	
60–79	619 6.51%	15 5.73%	
80–99	38 0.40%	0 0%	
**Gender**			0.19
Female	5909 62.08%	173 66.03%	
Male	3609 37.92%	89 33.97%	
**Exposure**			
**To a colleague at work**			0.02
No	6344 75.36%	161 68.51%	
Yes	2074 24.64%	74 31.49%	
**To a patient at work**			< 0.01
No	7114 84.46%	163 69.36%	
Yes	1309 15.54%	72 30.64%	
**In the private domain**			< 0.01
No	7728 89.81%	181 76.05%	
Yes	877 10.19%	57 23.95%	
**Recent travel outside Bavaria**			0.19
No	363 26.02%	19 33.93%	
Yes	1032 73.98%	37 66.07%	
**Other exposure**			0.52
No	7944 92.32%	217 91.18%	
Yes	661 7.68%	21 8.82%	

Among the positive test results, 74 (31.49%) belonged to clients who reported an exposure to a colleague at work followed by exposure to a patient at work 72 (30.64%), and exposure to a COVID-19 case in the private domain 57 (23.95%). From the clients tested, 1069 provided information about their recent travel history of which 37 (66.07%) tested positive for SARS-CoV-2. The majority of travel-related exposures were reported in February 2020 (9.07%), March 2020 (53.13%), August 2020 (5.71%), and January 2021 (6.64%) (see Additional file 1). The number of asymptomatic clients not reporting at least one symptom but testing positive for SARS-CoV-2 was 82 (32.28%). The most commonly reported symptoms in the first wave and second waves were sore throat (n = 1542), rhinorrhea (n = 1429), and cough (n = 1395). Further reported symptoms are listed in Table 2.

**Table 2:** Test results of clients based on clinical characteristic

Client characteristics	Tested negative	Tested positive	p-value
**Yes/No symptoms**			< 0.01
No	6558 70.94%	82 32.28%	
Yes	2687 29.06%	172 67.72%	
**Symptoms**			
**Fever**			< 0.01
No	8167 94.52%	165 68.75%	
Yes	470 5.44%	75 31.25%	
**Cough**			< 0.01
No	7403 85.23%	132 54.10%	
Yes	1283 14.77%	112 45.90%	
**Dyspnea**			0.11
No	8355 97.14%	228 95.40%	
Yes	246 2.86%	11 4.60%	
**Headache**			< 0.01
No	4268 83.88%	64 43.54%43.54%	
Yes	820 16.12%	83 56.46%	
**Sore throat**			< 0.01
No	7202 82.99%	173 72.38%	
Yes	1476 17.01%	66 27.62%	
**Rhinorrhoea**			< 0.01
No	7328 84.63%	142 59.17%	
Yes	1331 15.37%	98 40.83%	
**Anosmia/Ageusia**			< 0.01
No	6376 98.14%	129 83.77%	
Yes	121 1.86%	25 16.23%	
**Hemoptysis**			0.06
No	428 99.53%	29 96.67%	
Yes	2 0.47%	1 3.33%	
**Phlegm**			0.81
No	334 71.67%	23 69.70%	
Yes	132 28.33%	10 30.30%	
**Chest pain**			0.05
No	349 73.17%	19 57.58%	
Yes	128 26.83%	14 42.42%	
**Otalgia**			0.87
No	402 83.75%	28 84.85%	
Yes	78 16.25%	5 15.15%	
**Wheezing**			0.88
No	396 90.83%	27 90.00%	
Yes	40 9.17%	3 10.00%	
**Arthralgia**			< 0.01
No	398 81.89%	20 54.05%	
Yes	88 18.11%	17 45.95%	
**Myalgia**			0.01
No	332 66.94%	18 45.00%	
Yes	164 33.06%	22 55.00%	
**Fatigue**			0.16
No	201 40.61%	10 28.57%	
Yes	294 59.39%	25 71.43%	
**Confusion**			0.88
No	419 96.10%	29 96.67%	
Yes	17 3.90%	1 3.33%	
**Nausea/Emesis**			0.24
No	394 86.21%	26 78.79%	
Yes	63 13.79%	7 21.21%	
**Diarrhea**			0.18
No	394 80.74%	25 71.43%	
Yes	94 19.26%	10 28.57%	
**Conjunctivitis**			0.54
No	414 95.83%	29 93.55%	
Yes	18 4.17%	2 6.45%	
**Eczema**			0.81
No	432 97.30%	28 96.55%	
Yes	12 2.70%	1 3.45%	
**Lymphadenopathy**			0.17
No	410 92.97%	25 86.21%	
Yes	31 7.03%	4 13.79%	

### Age distribution and gender of clients

Among the 6989 clients during the overall period, the median age of the clients was 34 (IQR 26–48 ). Of the clients, 9.79% of the clients belonged to the age group 0–19, 51.73% belonged to the age group 20–39, 31.60% to the age group 40–59 and 6.49% to the age group 60–79.

**Fig. 1:**
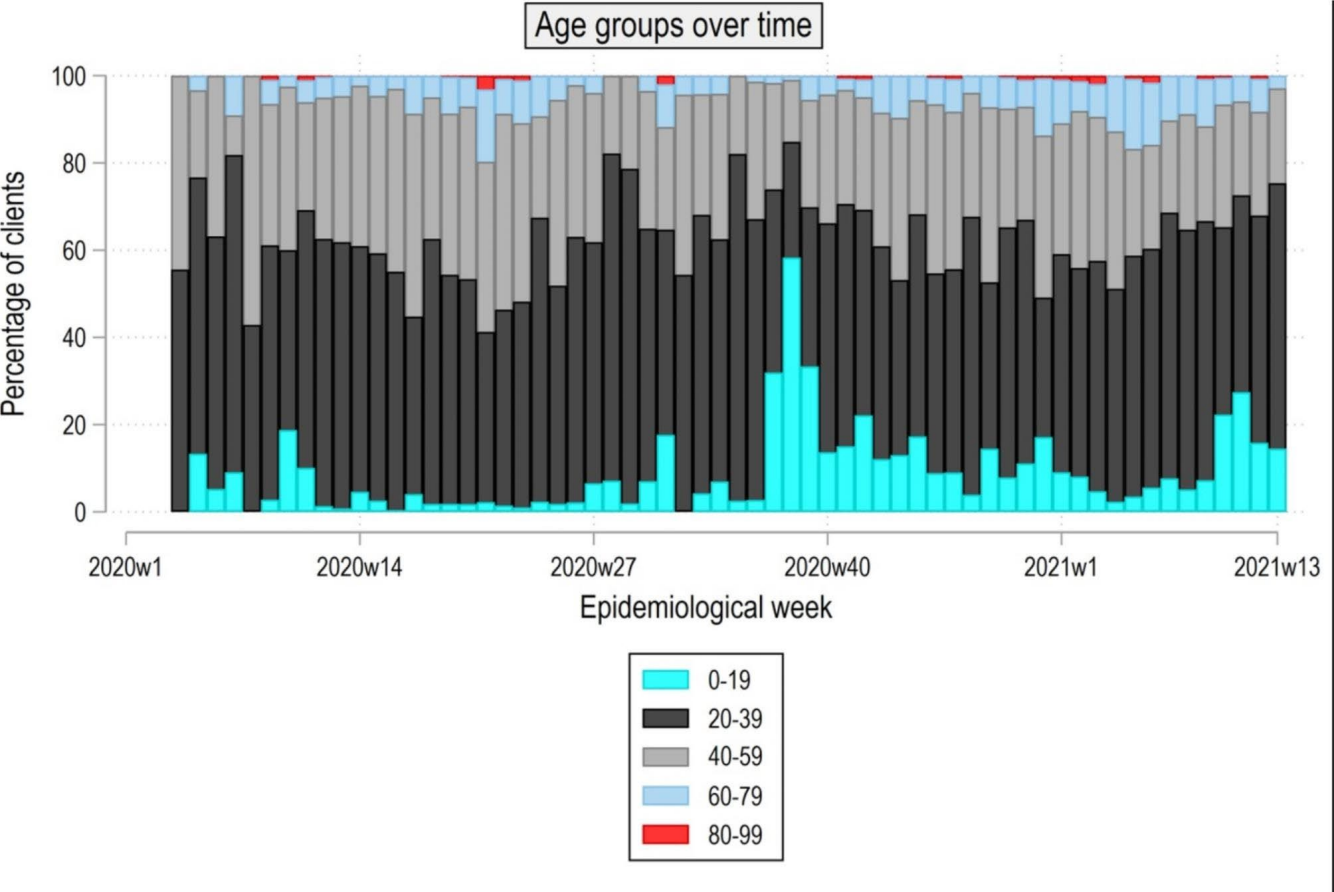
Age distribution of clients at the CTU per epidemiological week

The age distribution of clients at the CTU per epidemiological week can be seen in Fig. 1. On examination of the age of clients, divided into 19-year age groups, over time, the age group 20–39 and 40–59 years show comparatively high test proportions over the entire observed period (see Fig. 1). The testing numbers of the age group 0–19 years sharply peaked up to 58% in week 38 of 2020 with testing numbers remaining high thereafter. Age group 20–39 years accounted for the largest share of test numbers, only sporadically being overtaken in a total of 4 weeks. The increase in testing numbers of ages 60–79 roughly coincides with pandemic waves 1 and 2. Age group 80 and above, accounting for the smallest share in tests, showed little fluctuation over time.

### Reported exposures

**Fig. 2:**
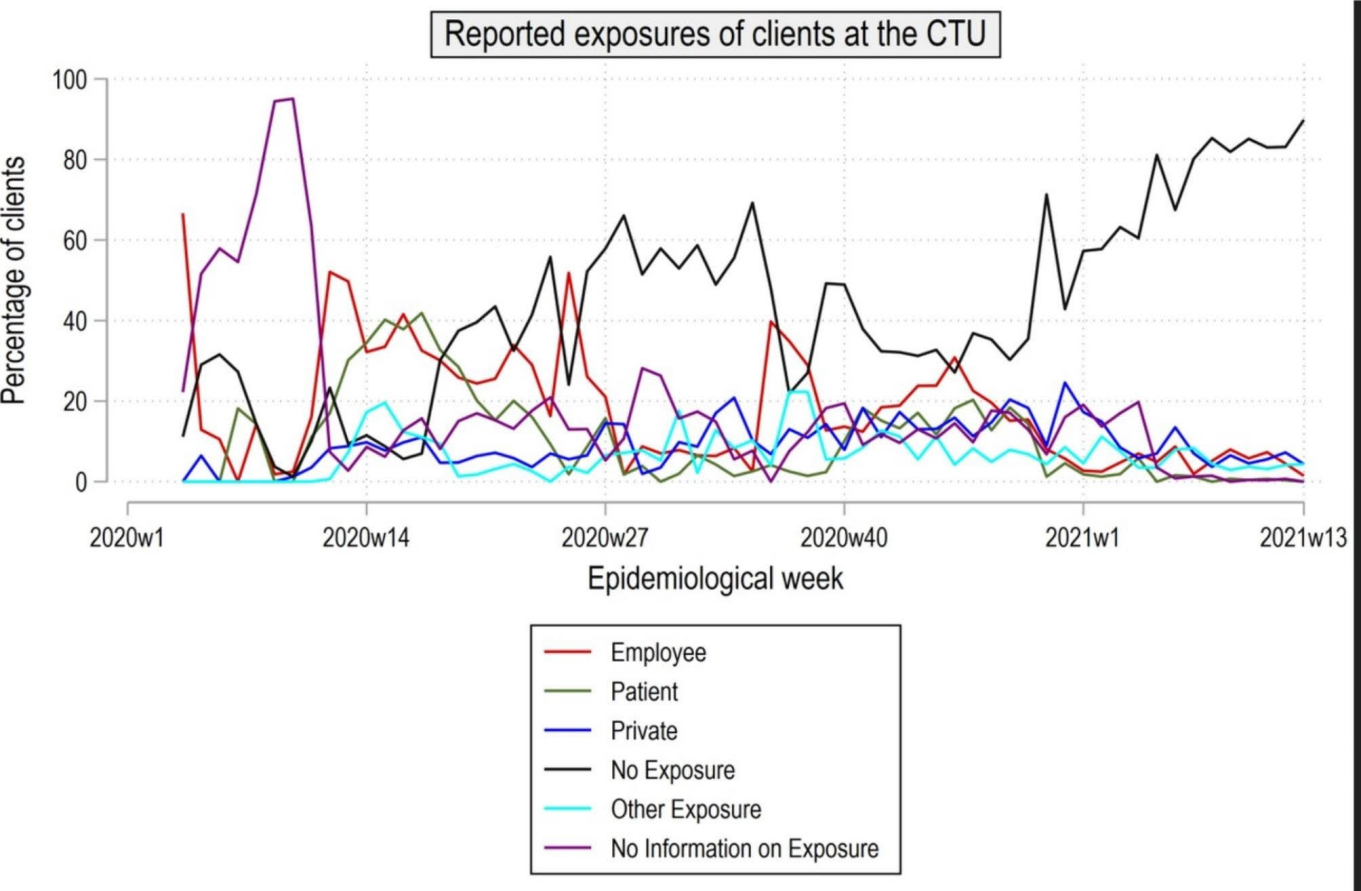
Exposure of clients at the CTU per epidemiological week

Figure 2. shows the exposure of clients at the CTU per epidemiological week. The proportion of clients with risk contacts at their place of work significantly increased in weeks 4 and 12 of 2020, showing a substantial rise in weeks 25 and 34 of 2020 followed by a consecutive decline (See Fig. 2). Exposure of clients to a positive index case in the private environment was frequently reported in the overall period. Amongst all exposures reported, clients associated with positive patients showed an increase in week 17 of 2020. However, an enhanced decline can be seen from week 24 to week 36 of 2020. Clients reported not to have had any exposure to appositive index cases more often after week 18 of 2020, with a consistent increase towards the end of the observed period until week 13 of 2020.

### Reported symptoms

**Fig. 3:**
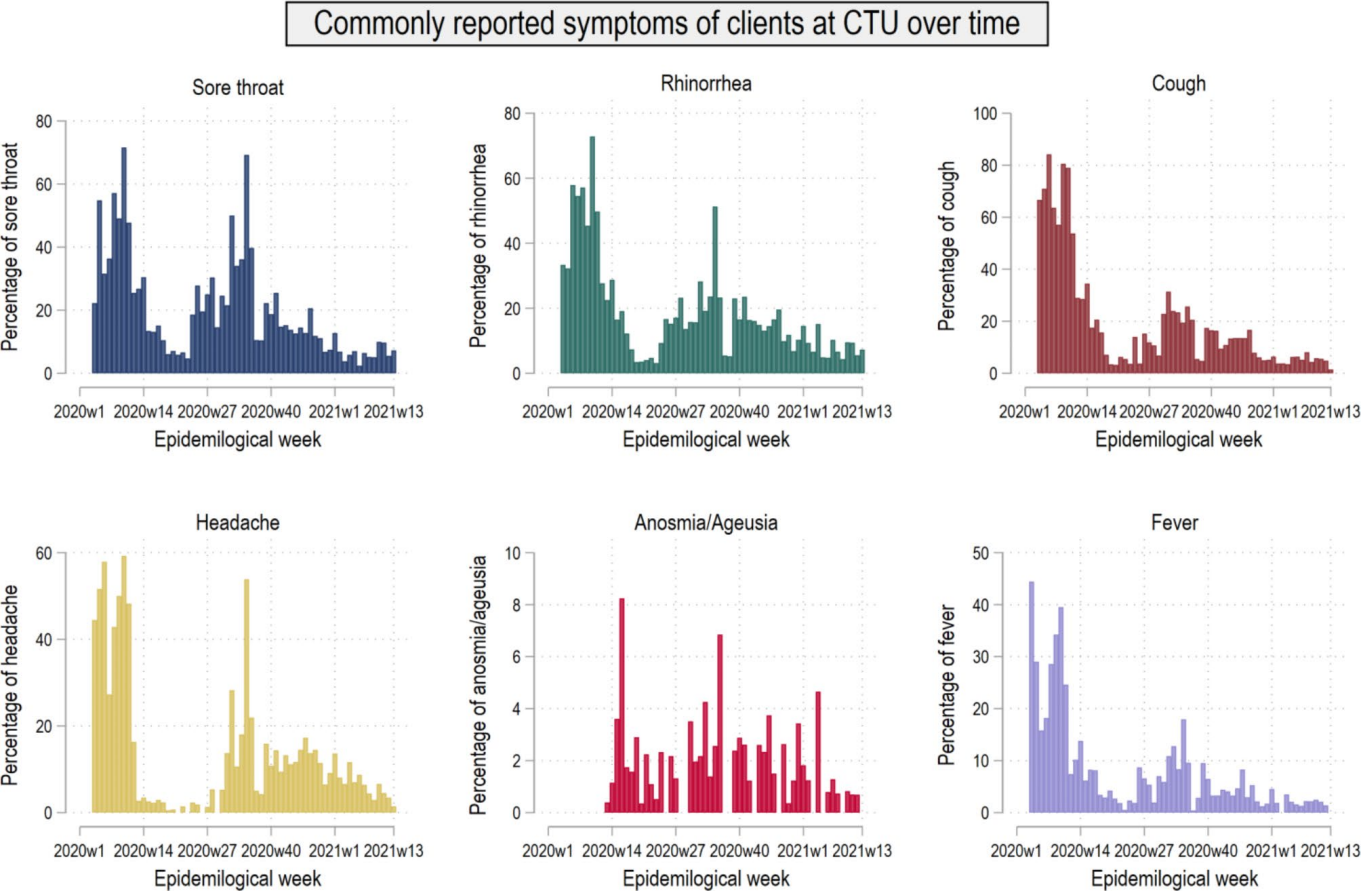
Commonly reported symptoms of clients at the CTU over time

Figure 3. shows commonly reported symptoms of clients at the CTU over time. A greater proportion of clients reported symptoms in the early weeks until week 15 of 2020 in the first wave, which was less frequently presented in the second wave. Similar to the first wave the reporting rates of sore throat (70%), headache (53%), rhinorrhoea (50%), anosmia, and ageusia (6.8%) were increasing at the beginning of the second wave.

### Breakpoint modeling of client numbers and 7-day incidence per 100,000 inhabitants in Bavaria over time

**Fig. 4:**
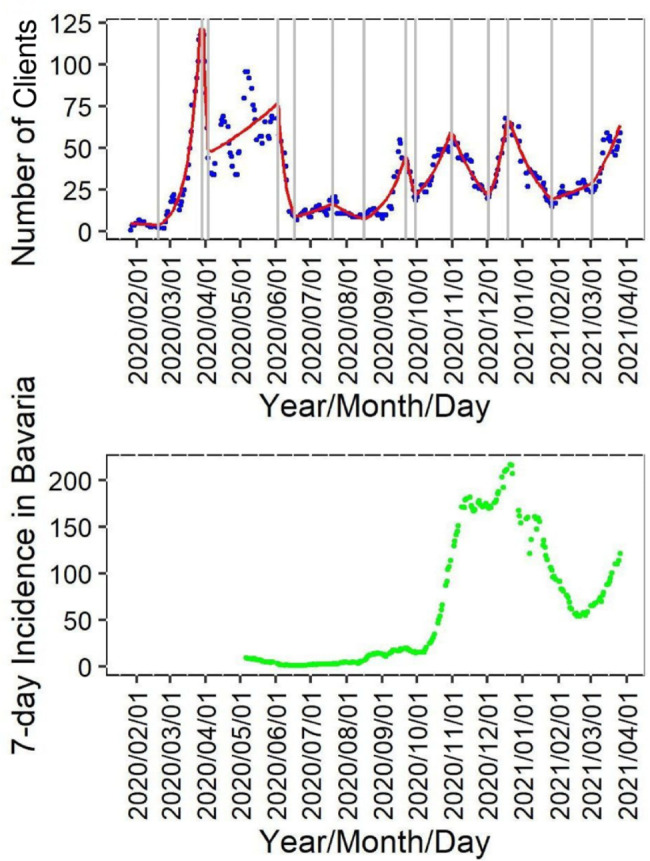
Client numbers in the CTU and COVID-19 incidence in Bavaria over time. 7-day moving average of daily client numbers (blue) and 7-day incidence per 100,000 inhabitants in Bavaria since start of recording (green), negative binomial model (red), breakpoints (grey). Note: The 7-day incidence per 100,000 inhabitants in Bavaria is adapted from the COVID-19 Incidence report by the Robert Koch Institute online database [12]

In the breakpoint analysis of 7-day moving averages of daily client numbers, a negative binomial model with 14 break points was found to be most suitable, measured in terms of R^2 and AIC. Breakpoints were thus set on 20 February 2020, 29 March 2020, 03 April 2020, 02 June 2020, 17 June 2020, 20 July 2020, 16 August 2020, 21 September 2020, 29 September 2020, 31 October 2020, 02 December 2020, 18 December 2020, 26 January 2021, and 01 March 2021. The timeframe of week 5 to week 26 of 2020 saw high fluctuations of patient numbers, which are only partially represented by the model (see Fig. 4). In contrast, the section between week 34 of 2020 and week 4 of 2021 shows less sharp rises and the numbers can be well fitted by the breakpoint model.

Also shown in Fig. 4 are the 7-day incidence rates in the general population of Bavaria since its beginning from May 2020 [12]. The incidence rates reached more than 200 per 100,000 inhabitants during January 2021.

### Correlation between public health measures and patient numbers and test positivity

**Fig. 5:**
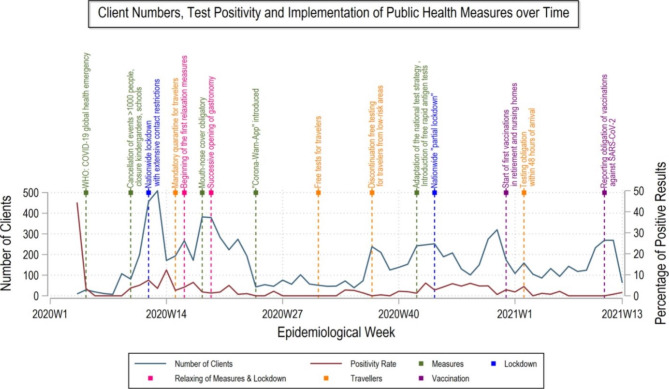
Timeline of client numbers and proportion of positive test results with the adoption of public health measures over time

Figure 5- shows Timeline of client numbers and proportion of positive test results with the adoption of public health measures over time. The CTU started with the highest peak of test positivity rate in week 4 of 2020. The beginning of the first COVID-19 wave in Munich was around week 10 of 2020, where we could see exponential growth with daily testing numbers reaching 500 around week 13 of 2020. At the same time, infection hygiene measures to limit interpersonal contacts were tightened, with the cancellation of events with more than 1000 participants and the closure of schools and kindergartens. This extended to a comprehensive lockdown in week 13 of 2020 [13, 14]. During the lockdown, testing rates and test positivity rates were high at the CTU with test positivity reaching 10% in week 14 of 2020. The first wave ended in week 20 of 2020, with control measures restricting contact continuing to loosen up gradually in week 19 of 2020 [15]. In the period from week 21 of 2020 to week 37 of 2020, testing numbers continued to decrease with test positivity rates of less than 5%. At the same time, free tests for travellers arriving from abroad were made available in week 31 of 2020 [16]. At the beginning of autumn from week 38 of 2020 onwards, there was a fluctuation in the number of tests administered with about 250 as a weekly average and test positivity rates of less than 2% at the CTU. In week 42 of 2020, the national testing strategy was adapted and free antigen tests were introduced in nursing homes and hospitals [17]. Further was a slight increase in client testing numbers followed by a nationwide partial lockdown in week 45 of 2020 [18]. Following the first week of 2021, there were shifts in testing numbers, with the weekly number of tests not exceeding 200. Around week 10 of 2021, the number of tests increased steadily, followed by a reduction toward the end of the CTU with a test positivity rate of 3%.

## Discussion

This is a retrospective observational study on the clinical presentation of COVID-19 in the first testing unit in Munich and includes data from 6989 clients. We sought to describe external and internal influencing factors on a COVID-19 testing station in a particularly space- and time-sensitive context.

### Research findings and comparison with literature

As seen from our analysis, the demographic characteristics of the clients were mostly young and female, as compared to the German population [19]. As expected, health care workers and students were subjected to frequent testing due to case clusters in hospitals and schools,, which were amongst the institutions that referred clients to the CTU. Later the CTU was associated with a school cohort consequently causing an increase in test numbers in the age group 0–19 early in the second wave of the pandemic (see Fig. 1).

Numerous symptoms were reported by the clients over the observed period, which are summarized in Table 2. The most commonly reported symptoms relate to infections of the airway tract (sore throat, rhinorrhoea, cough) and showed comparatively high association with SARS-CoV-2 positivity. Dyspnea was reported by 4.60% of the clients who tested positive but was not associated with SARS-CoV-2 infection. Moreover, 25 clients (16.23%) with anosmia and ageusia reported SARS-CoV-2 infection, which was lower as compared to a study in the US outpatient setting where more than one third of the individuals with anosmia and ageusia tested positive for SARS-CoV-2 infection [20]. Notably, while rather unspecific flu-like symptoms were reported from week 4 of 2020 on, anosmia and ageusia only started to appear in client records from week 13 on. It has to be assumed that the perception of COVID-19 specific symptoms in individuals changed over time under the influence of media coverage and official case definitions, which only began to include anosmia and ageusia as specific symptoms relatively late [21]. Gastrointestinal symptoms (diarrhoea, nausea and emesis) showed a lower correlation with SARS-CoV-2 infection.

Clients reported symptoms more frequently in the first wave as compared to the second wave (see Fig. 3). This was likely not caused by changes in characteristics of the virus, but because clients were pre-selected and triaged for symptoms and exposures during the first period of operations of the CTU. This illustrates a balance between efficient use of limited resources while at the same time broadening the availability of tests later on. Notably, symptoms that are widely considered COVID-19 related were still reported after triage was lifted, although the proportion of asymptomatic clients rose until the end of the reported period. Since COVID-19 waves 1 and 2 coincided roughly with the common influenza-virus seasons in Europe, there might be an overlap of COVID-19 and influenza symptoms resulting in clients testing for both infections [20].

In addition to the symptoms, having undergone specific exposures was also associated with testing positive for SARS-CoV-2. Test positivity was higher in those exposed to individuals with COVID-19 at work (31.49%).Although many public health measures (e.g., travel restrictions, quarantine measures) aimed at reducing the risk of importing cases from abroad, we could not conclude that travelling abroad was a particular risk factor for SARS-CoV-2 infection. Specifically at the beginning of the pandemic in Germany during the winter holidays in February of 2020, people were returning from commonly visited skiing areas in Austria [22]. Strong beer festivals in Bavaria around week 11 of 2020 could also be an important source of SARS-CoV-2 infection [8]. At the same time, we could also observe an increased number of travel returnees at the CTU in February (9.07%) and March 2020 (53.13%) (see Additional file 1). As the pandemic progressed, the number of clients reporting information about travel was much reduced as compared to the early stages of the pandemic. It could be assumed that the pandemic has created an aversion towards traveling. Here again, pre-selection bias by triage for specific exposures has to be taken into consideration.

### German governmental measures to combat the pandemic

Infection rates were and continue to be an important indicator in controlling a pandemic. A critical decision is how and when to implement community-level non-pharmaceutical interventions (NPIs). Public health authorities must choose an appropriate set of NPIs for implementation when a pandemic starts and constantly adapt them with changes in knowledge, infection rates, available resources, and public attitude. On the basis of the circumstances in the relevant jurisdiction, governing authorities, and state and municipal officials make these judgments [23].

Germany established a central coordinating and decision-making body as a federal state, and crisis management decisions were taken with the participation of leaders of its regional state governments [24].

With the beginning of the first wave in Germany in the 10th week of 2020, there was an exponential growth in the testing numbers around week 11 of 2020 that can be related to the growing transmission through regional festivals and public gatherings [25]. A study by Frank et al. reported that infections were also emerging with the arrival of people from China and ski returns from Italy and Austria [26]. Notably, we could also observe an increasing test positivity rate (3%) at the CTU. The government began canceling events with more than 1000 expected participants along with the closure of kindergartens and schools in the 11th week of 2020 [13, 14]. With rising case numbers in Germany in weeks 12 and 13, we also observed an increased influx of clients, and therefore an outdoor tent was set up at the CTU in order to increase testing capacities and reduce the risk of nosocomial infection between clients.

Likewise, public restrictions were increased in short sequences over the time frame from the end of March to the beginning of June 2020. With the advent of the summer season, and an increase in environmental temperatures and outdoor activities of the general population, a reduction in case numbers could be seen; restrictions were thereon reduced. Likewise, we could notice a reduction in the demand for testing at the CTU. Despite some relaxations, certain measures remained in place over the summer months, including mandatory face mask-wearing in public places and social distancing. The “Corona-Warn-App” was deployed in Germany around the 25th week of 2020 with the goal to ensure a cautious, steady approach to detecting and monitoring infections. It was intended to indicate to its users close contacts to other app users that then turned out to be SARS-CoV-2 positive [27]. However, later in summer 2020, travel return activities and the introduction of the Bavarian testing strategy, which enabled indication-free testing, again increased the testing numbers at the CTU, but now increasingly among non-symptomatic individuals.

The second wave of COVID-19 in Munich started at the end of September 2020, peaked in December 2020, and was ongoing until March 2021. To mitigate high infection rates, a nationwide lockdown, free antigen tests, and night curfews were introduced in the upcoming fall and winter seasons of 2020 [17, 18]. The 52nd week of 2020 saw the beginning of vaccinations in retirement homes to combat the pandemic [28].

According to our analysis of client numbers at the CTU, we are observing an association between certain public health measures and absolute client numbers. It should be emphasized that the test positivity rate at the CTU exhibited very minor fluctuations in the later stages of the pandemic irrespective of the testing numbers and adoption of public health measures at different stages during the study period. If the test positivity rates remain stationary regardless of the dynamics in testing numbers, it may lead to the assumption that changes in the public health measure do not result in a more efficient case detection but just in a fluctuation of absolute case numbers that merely depend on the absolute numbers of tests that are being executed in a given facility. In other words, the absolute reported case numbers in a given public health context would be much more linked to changes in the utilization by the population of testing offers, which in turn is influenced by invigorated public health measures, as compared to true fluctuations in the epidemiological situation. This systematic effect on perceived needs, accessibility and acceptability has to be considered in the interpretation of observed dynamics in a pandemic, if the models are mainly based on case numbers as notified to public health authorities.

### Strength of our study

Our ability to offer data from the first testing unit in Germany and a comprehensive dataset without debarring any information from the start of the pandemic until March 26 of 2021 is the main strength of this study. Our results aid in understanding the progression of the pandemic, and the effects of early public health interventions with regard to dynamics in client numbers and test positivity rates. In our opinion, our data can support both public health authorities as well as epidemiologists that are engaged in model development, in aligning public health measures with surveillance data.

### Limitations

The external validity and representativeness of our results have limitations. In addition, the client population is concentrated in certain occupational categories, including employees, retirees in nursing homes, and healthcare professionals. Another limitation is that clients may have exaggerated symptoms in order to obtain a test, especially when testing was restricted to rigorous triage criteria at the beginning of the pandemic. Increasing yet unbalanced knowledge in the general public on the virus characteristics as well as vaccinations may have led to a false sensation of security and may have impeded client testing. Since this study is cross-sectional, we are unable to report on the results of possible follow-up testing in clients that may have tested negative at first presentation.

## Conclusion

This analysis provides information on the very first phase of the COVID-19 pandemic in Germany. Over the course of 14 months, we were able to analyze data on 6989 clients. Our data demonstrate that testing was initially confined to risk groups and travel returnees as a result of triage measures. Public health measures such as contact restrictions and lockdowns are closely related to testing numbers and test positivity rates, for example, the first lockdown in 2020 led to reduced utilization of services and hence to lower case confirmations, which in turn led to lower reported case numbers. It is important to recognize these phenomena when using reported case numbers for guidance in the implementation of public health measures or when evaluating the efficacy of these. Our study is meant to inform stakeholders and give insight into these potential side effects of public health measures.

## Electronic supplementary material

Below is the link to the electronic supplementary material.

Supplementary Material 1

## Data Availability

Datasets used and/or analyzed during the current study are available upon reasonable request from the corresponding author.
